# Emodin triggers cuproptosis to suppress hepatocellular carcinoma via SLC7A11/FDX1 axis

**DOI:** 10.3389/fonc.2026.1756712

**Published:** 2026-03-25

**Authors:** Yantong Chen, Yanmei Liu, Jianwen Huang

**Affiliations:** 1 State Key Laboratory of Traditional Chinese Medicine Syndrome, Guangzhou University of Chinese Medicine, Guangzhou, China; 2 School of Basic Medical Sciences, Guangzhou University of Chinese Medicine, Guangzhou, China; 3 The Research Center of Basic Integrative Medicine, Guangzhou University of Chinese Medicine, Guangzhou, China; 4 Stem Cell and Traditional Chinese Medicine Regenerative Medicine Team, Guangzhou University of Chinese Medicine, Guangzhou, China; 5 Guangdong Academy of Sciences Foshan Industrial Technology Research Institute, Foshan, Guangdong, China

**Keywords:** cuproptosis, emodin, FDX1, GSH, hepatocellular carcinoma, SLC7A11

## Abstract

**Background:**

Hepatocellular carcinoma (HCC) is a lethal malignancy with limited therapeutic options, necessitating novel treatment strategies. This study investigates the potential of emodin, a natural anthraquinone, to suppress HCC by inducing cuproptosis, a newly identified form of regulated cell death.

**Method:**

The anti-tumor effects of emodin were evaluated both *in vitro*using HCCLM3 cells and *in vivo* using nude mouse xenograft models. A series of assays were employed to assess cell proliferation, apoptosis, intracellular copper ion levels, and glutathione (GSH) levels. The expression of key proteins (FDX1, SLC7A11, GPX4) was examined by Western blot and immunohistochemistry. Furthermore, bioinformatics analysis was conducted to predict the interaction between emodin and cuproptosis-related proteins. Crucially, FDX1-knockdown (si-FDX1) and pharmacological inhibition experiments using the copper chelator tetrathiomolybdate (TTM) were performed to establish causality and pathway specificity.

**Result:**

Emodin treatment dose-dependently inhibited HCC cell proliferation, promoted apoptosis, elevated intracellular copper levels, and reduced GSH. Mechanistically, emodin upregulated the expression of FDX1 while downregulating SLC7A11 and GPX4. Molecular docking analysis supported the binding capability of emodin to these core proteins involved in cuproptosis. Most importantly, FDX1 knockdown abolished emodin-induced copper accumulation and rescued cell death. Furthermore, the cytotoxicity was specifically reversed by the cuproptosis inhibitor TTM, but not by ferroptosis or apoptosis inhibitors, confirming the specificity of the death pathway.

**Conclusion:**

Our findings demonstrate that emodin triggers cuproptosis in HCC via the SLC7A11/FDX1 axis. This reveals a novel mechanism underlying emodin’s anti-tumor activity and highlights its promise as a therapeutic agent for HCC, particularly in SLC7A11-overexpressing subtypes, with potential to enhance combination therapies and overcome drug resistance.

## Introduction

Hepatocellular carcinoma (HCC) remains the most prevalent primary liver malignancy worldwide, and its increasing incidence and mortality rates pose critical public health challenges. According to projections from the International Agency for Research on Cancer (IARC) of the World Health Organization (WHO), new global HCC cases could reach 1.4 million by 2040, resulting in approximately 1.3 million associated deaths worldwide (1). Even though we have made progress with new ways to treat advanced liver cancer using medicines that target the cancer cells directly, and medicines that boost the immune system to fight the cancer, these medicines do not work well for everyone. Moreover, there is the potential for them to engender deleterious side effects, not to mention the exorbitant expense they can incur. This means that we need to find new ways of treating people with liver cancer.

Emodin (EMO), a naturally occurring anthraquinone derived from various medicinal herbs such as *Rheum palmatum and Polygonum cuspidatum*, has demonstrated broad-spectrum anti-tumor efficacy in multiple cancer models, including HCC (2). Related anthraquinone compounds, such as aloe-emodin, have also shown promising pharmacological effects. Recent evidence demonstrates that aloe-emodin alleviates myocardial intracellular calcium homeostasis imbalance induced by high-fat diet through the PRMT1/CaMKII signaling pathway, highlighting the therapeutic potential of anthraquinones in metabolic and cardiovascular diseases (3). Emodin has been found to reduce the amount of glutathione (GSH) inside cells (4), which suggests that it may be involved in regulating cuproptosis. It is a new type of cell death caused by copper-dependent issues in the mitochondria and the accumulation of specific proteins within these cells. This makes emodin an exciting potential therapeutic for targeting copper-dependent oncogenic signaling pathways.

Cuproptosis is initiated by the binding of pathological copper accumulation to lipoylated TCA cycle proteins, which directly disrupts mitochondrial metabolism, induces proteotoxic stress and results in cell death (5). There is emerging evidence that supports the induction of cuproptosis as a potential anti-tumour strategy (6). The core regulators are ferredoxin 1 (FDX1), which catalyzes the reduction of Cu²^+^ to the toxic Cu^+^ form and upregulates protein lipoylation to create copper-binding sites (5, 7), and GSH, a cytosolic copper chelator that inhibits cuproptosis (8, 9). Therefore, the concentration of intracellular GSH constitutes an essential negative regulator of copper homeostasis.

Glutathione peroxidase 4 (GPX4) and the cystine/glutamate antiporter SLC7A11, the functional subunit of system Xc⁻, constitute important regulators of cellular glutathione (GSH) homeostasis. GPX4 reduces glutathione disulfide (GSSG) in order to regenerate GSH, while SLC7A11 facilitates the import of cystine, which is essential for *de novo* GSH synthesis (10, 11). The SLC7A11-GPX4-FDX1 axis constitutes the core ‘copper toxicity amplification and antioxidant defense’ system that influences GSH metabolism. Therefore, we hypothesize that emodin induces copper-toxicity-mediated apoptosis by disrupting the SLC7A11-GPX4-FDX1 regulatory cascade.

This study integrates *in vitro* and *in vivo* approaches to systematically investigate how emodin induces cuproptosis in HCC cells. Our aim is to clarify the roles of SLC7A11, FDX1 and GPX4 in order to gain mechanistic insights into emodin’s anti-tumour effects and to support its potential as a therapeutic agent for HCC.

## Materials and methods

### Reagents

Emodin (purity 99.22%, S2295), Phosphatase Inhibitor Cocktail (B15002), Tween-80 (S6702), and PEG300 (S6704) were obtained from Selleck (USA). CCK-8 Kit (C0038), GSH and GSSG Assay Kit (S0053), Protease Inhibitor Cocktail for General Use (P1005), and Eosin Staining Solution (C0109) were purchased from Beyotime (China). Annexin V-FITC/PI Apoptosis Kit (E-CK-A211) and Cell Copper Colorimetric Assay Kit (E-BC-K775-M) were obtained from Elabscience (China). BCA Protein Assay Kit (FD2001) and RIPA Lysis Buffer (FD009) were purchased from FUDE Biological Technology (China). Dimethyl sulfoxide (DMSO, cell culture grade, D8371) was purchased from Solarbio (China). 4% Paraformaldehyde (DF0135) was purchased from Leagene (China). DAB Kit (G1212-200T) and Hematoxylin Staining Solution (G1004) were obtained from Servicebio (China). Immunohistochemistry Kit (SA1023) and Sodium Citrate Buffer (pH 6.0, AR0024) were purchased from Boster (China). Primary antibodies against SLC7A11 (DF12509), GPX4 (DF6701), and β-actin (T0022) were obtained from Affinity (USA), and the antibody against FDX1 (ab108257) from Abcam (UK). Tetrathiomolybdate (TTM, 10 μM, Sigma-Aldrich, Cat# 323446), Ferrostatin-1 (Fer-1, 2 μM, Selleck, Cat# S7243), Z-VAD-FMK (20 μM, Selleck, Cat# S7023).

### Cell culture

HCCLM3 cells (ZQ0023, STR-verified) were purchased from ZQXZ Bio (China) and cultured in DMEM (C11995500BT, Gibco, USA) supplemented with 10% fetal bovine serum (FBS, 164210, Procell, China) and 1% penicillin-streptomycin (C10010500BT, Gibco, USA) in a 5% CO_2_ incubator at 37 °C. All experiments were performed using cells in the logarithmic growth phase.

### Cell transfection

Small interfering RNA (siRNA) targeting human FDX1 (si-FDX1) and a non-targeting negative control siRNA (si-NC) were synthesized by GenePharma (Shanghai, China). HCCLM3 cells were transfected with siRNA using Lipofectamine 3000 (Invitrogen) according to the manufacturer’s protocol. Knockdown efficiency was validated at both mRNA and protein levels by RT-qPCR and Western blot 48 hours post-transfection. For functional assays, cells were treated with emodin (20 μM) 24 hours after siRNA transfection.

### CCK-8 assay

Cell proliferation was assessed using the CCK-8 assay. HCCLM3 cells were seeded into 96-well plates at a density of 5 × 10³ cells/well. After 24 h, the medium was replaced with DMEM containing various concentrations of emodin for 24 or 48 h. Then, 10% CCK-8 reagent was added and incubated for 2 h. Absorbance was measured at 450 nm using a microplate reader (Agilent, USA), and cell viability was calculated.

### Flow cytometry

Apoptosis was analyzed by Annexin V-FITC/PI staining. HCCLM3 cells were seeded in 6-well plates at 4 × 10^5^ cells/well and treated with different concentrations of emodin for 24 h. Cells (including supernatant and adherent cells) were collected by centrifugation (2, 000 rpm, 5 min, 4 °C), resuspended in binding buffer, and stained with Annexin V-FITC and PI for 15 minutes at room temperature in the dark. After filtration through a 70 μm cell strainer, samples were analyzed using a BD flow cytometer. Data were processed with FlowJo software; Annexin V^+^/PI⁻ cells were identified as early apoptotic, and Annexin V^+^/PI^+^ cells as late apoptotic or necrotic.

### Bioinformatics analysis

The prognosis analysis included Overall Survival (OS) and Disease-Free Survival (DFS) of patients with HCC, used data from the Cancer Genome Atlas Program (TCGA). The Kyoto Encyclopedia of Genes and Genomes (KEGG) analysis was performed using the bioinformatics database from STRING (cn.string-db.org) and HERB 2.0 (herb.ac.cn).

### Molecular docking

The AutoDock Vina software was employed to achieve molecular docking between emodin and FDX1, GPX4, and SLC7A11 proteins. The structures of the proteins were downloaded from the RCSB PDB protein structure database (www.rcsb.org), and the chemical structure of emodin was obtained from the PubChem platform (pubchem.ncbi.nlm.nih.gov). The PyMOL software was used to visualize the output of the results.

### Intracellular copper ion quantification

Cells (5 × 10^6^ per 10 cm dish) were treated with emodin for 24 h. After collection, cells were lysed in 100 μL lysis buffer per 1 × 10^6^ cells and incubated on ice for 10 min. Lysates were centrifuged (11, 000 rpm, 10 min, 4 °C), and supernatants were subjected to copper quantification using a colorimetric assay kit, following the manufacturer’s instructions. Absorbance at 580 nm was measured, and intracellular copper concentration was normalized to protein content measured by BCA assay.

### Glutathione (GSH and GSSG) assay

Cells were seeded in 6-well plates (4 × 10^5^ cells/well), treated with emodin for 24 h, and collected. Cells were weighed, and every 10 mg of cell mass was lysed in 30 μL Protein Removal Reagent M. After vortexing and incubation at 4 °C for 5 min, samples were centrifuged (10, 000 rpm, 10 min, 4 °C). For GSSG measurement, a portion of the supernatant was treated with GSH-removal reagent and incubated for 60 min. Then NADPH solution (0.5 mg/mL) was added, and the absorbance at 412 nm was recorded after 25 min at room temperature. GSH and GSSG levels were calculated and normalized to total protein concentration.

### Animal experiments

Thirty five male Balb/c nude mice (5 weeks old) were purchased from Cyagen (Guangzhou, China). All procedures were approved by the Animal Ethics Committee of Guangzhou University of Chinese Medicine (No. 20230525007). Mice were housed under standard conditions with free access to food and water. Due to the stable tumor formation and relatively consistent growth rate of HCCLM3 cells in subcutaneous transplantation models, HCCLM3 cells were selected for animal modeling. Each mouse was subcutaneously injected with 2.5 × 10^6^ HCCLM3 cells in the right dorsal flank. Tumor formation was monitored, and after 14 days, mice were randomly assigned to three groups based on initial tumor volume (n=10 each): model group (vehicle: 10% DMSO, 40% PEG300, 5% Tween-80, 45% ddH_2_O), emodin low-dose (10 mg/kg), and high-dose (20 mg/kg). The selected doses were based on previous studies demonstrating significant antitumor efficacy without observable toxicity in murine models (2, 11). Emodin or vehicle was administered once daily by gavage for 14 days. Tumor volume and body weight were recorded regularly. Tumors were collected at the end of treatment (**Supplementary Tables 1**, **2**).

### H&E staining

Tumor tissues were fixed in 4% paraformaldehyde, dehydrated, paraffin-embedded, and sectioned at 4 μm. After deparaffinization and hydration, sections were stained with hematoxylin, blued under running water, and counterstained with eosin. Following dehydration and mounting, sections were observed and photographed using a pathological imaging system.

### Immunohistochemistry

Paraffin-embedded tumor tissues were sectioned at 4 μm. After deparaffinization and hydration, antigen retrieval was performed using sodium citrate buffer (pH 6.0), followed by permeabilization in 0.5% Triton X-100 and blocking with 10% goat serum. Sections were incubated overnight at 4 °C with primary antibodies against FDX1, GPX4, and SLC7A11 (1:200 each). After washing, sections were incubated with 3% H_2_O_2_, followed by a broad-spectrum secondary antibody and SABC reagent. DAB chromogen was used for signal development, and sections were counterstained with hematoxylin, dehydrated, and sealed. Images were acquired under a microscope, and positive cells were quantified from at least four randomly selected high-power fields (400×).

### Western blot

Expression of FDX1, GPX4, and SLC7A11 was examined by Western blotting. Tissues or cells were lysed in RIPA buffer containing protease and phosphatase inhibitors. After centrifugation (11, 000 rpm, 15 min, 4 °C), protein concentrations were determined using a BCA assay. Equal amounts of protein were separated by SDS-PAGE and transferred to PVDF membranes. Membranes were blocked in 5% non-fat milk for 2 h, incubated overnight at 4 °C with primary antibodies (1:1, 000 for FDX1, GPX4, SLC7A11, and β-actin), and then incubated with HRP-conjugated secondary antibodies (1:15, 000) at room temperature for 1 h. Signals were visualized using a chemiluminescence system and quantified with Image J software.

### Statistical analysis

Statistical analyses were performed using SPSS 26.0. Normally distributed data were expressed as mean ± SD and analyzed by *One-way ANOVA*. Followed by *LSD* or *Dunnett’s T3* test. Non-normally distributed data were expressed as median (IQR) and analyzed using non-parametric methods. Significance levels were indicated as *P < 0.05, **P < 0.01, ***P < 0.001.

## Results

### Emodin induces apoptosis in HCCLM3 cells

**Figure 1:**
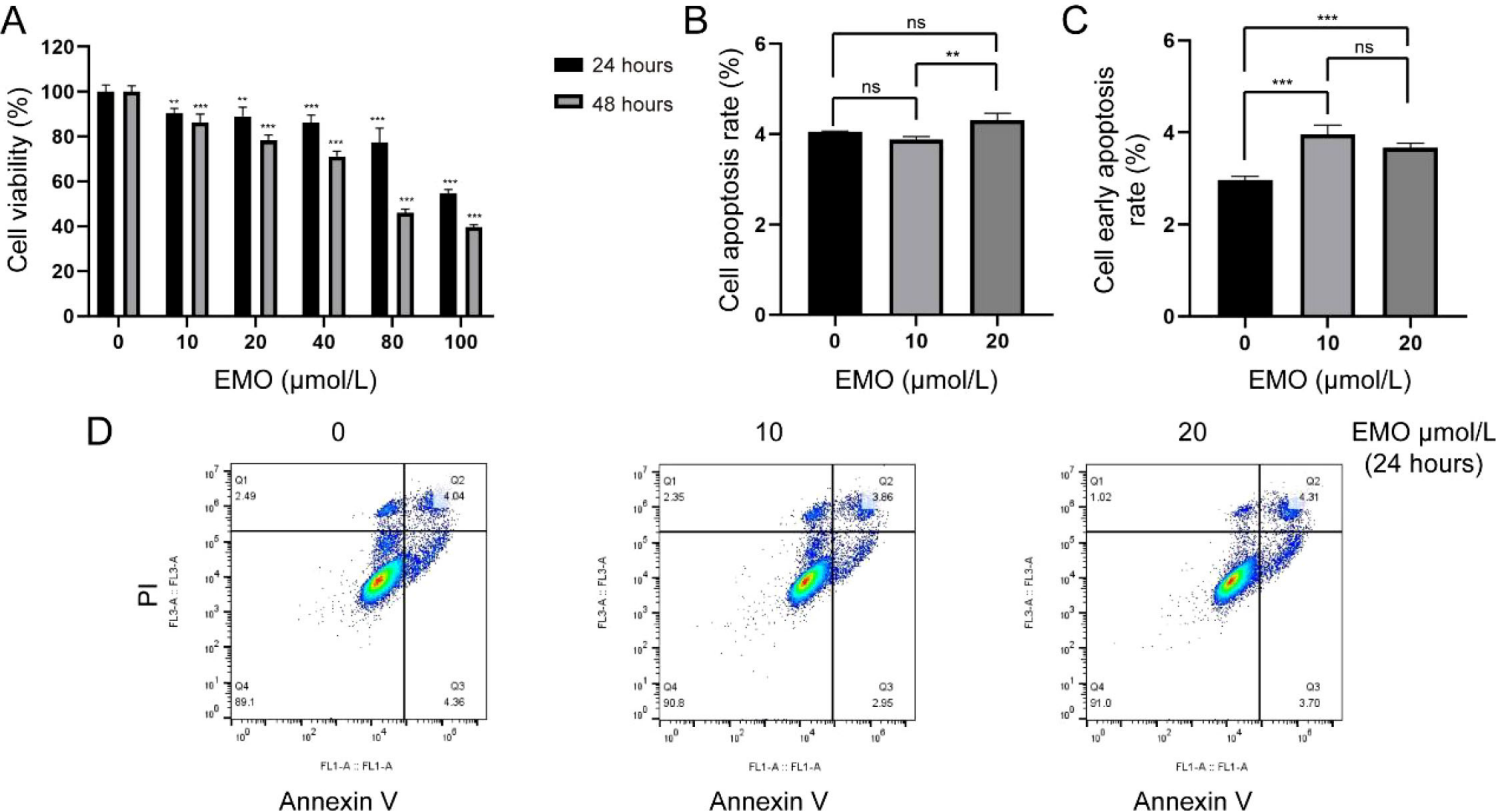
Emodin inhibited proliferation and promoted apoptosis in HCCLM3 cells. **(A)** Viability of HCCLM3 cells after intervention with different concentrations of emodin for 24 h (IC50 = 157.9, R^2^ = 0.7260) and 48 h (IC50 = 72.31, R^2^ = 0.9586). (n=4) Compared with the control group, ^**^P < 0.01, ^***^P < 0.001. The apoptosis level **(B)** and early apoptosis level **(C)** of HCCLM3 cells after intervention with different concentrations of emodin for 24 (h) (n=3) **(D)** Representative images of flow cytometry. ns, *P* > 0.05, ^**^P < 0.01, ^***^P < 0.001.

A total of 35 mice were injected, with 30 successfully establishing the tumor model. The tumor formation rate was 85.7%. To evaluate the direct effects of emodin on HCCLM3 cells, we first conducted CCK-8 assays and apoptosis analysis. The CCK-8 results showed that cell viability decreased in a concentration- and time-dependent manner following emodin treatment at both 24 and 48 h, compared with the control group (**Figure 1A**), suggesting that emodin may inhibit the proliferation of HCCLM3 cells. Based on these preliminary results, apoptosis assays employed emodin at concentrations of 0, 5, 10, 20 and 40 µmol/L (**Supplementary Figure 1**). we selected 0-20 μmol/L for subsequent apoptosis assays. Flow cytometry analysis revealed a marked increase in early apoptosis at 10 μmol/L, and a significant elevation in both early and total apoptosis at 20 μmol/L (**Figures 1B–D**), indicating that emodin promotes apoptosis in HCCLM3 cells. This indicates that metabolic stress precedes apoptotic determination, consistent with copper-induced death mechanisms.

### Associations among cuproptosis-related proteins and emodin in HCC

**Figure 2:**
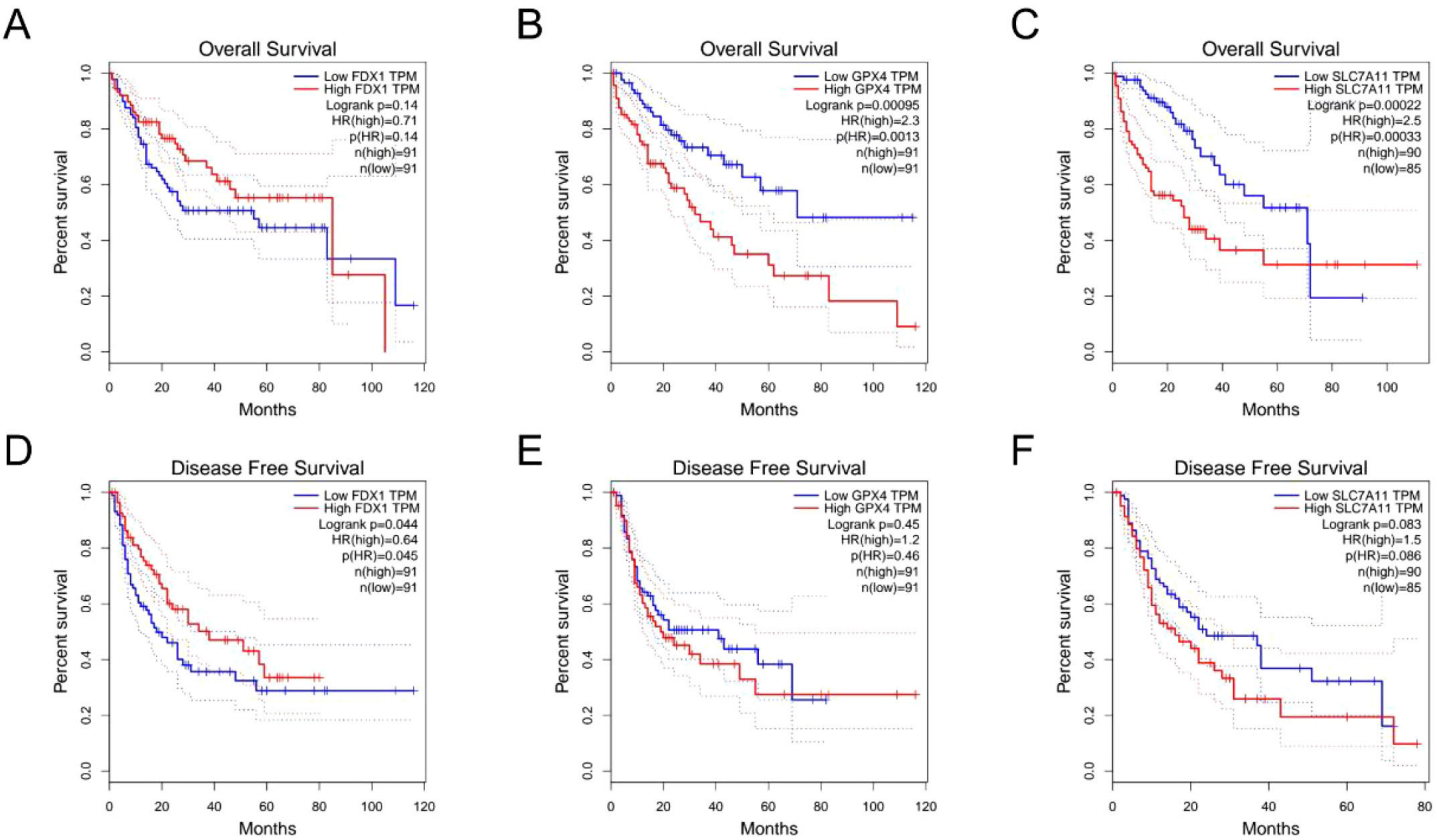
Cuproptosis-related proteins affect HCC prognosis and block with emodin. The prognostic prediction performances of FDX1, GPX4 and SLC7A11 for OS **(A–C)** and DFS **(D–F)** were assessed by Kaplan-Meler curve analysis through TCGA database.

We performed bioinformatics analysis to predict the effect of cuproptosis on HCC The high expression of FDX1 among HCC patients might have a higher DFS (**Figure 2D**), suggesting that it might reduce HCC recurrence; the low expression of GPX4 and SLC7A11 might leave a better prognosis (**Figures 2A–F**). To determine whether emodin influences cuproptosis, the KEGG enrichment analysis of FDX1 was performed. GPX4 and SLC7A11 showed that the genes were enriched in the glutathione metabolism pathway (FDR = 1.7E-43), HCC pathway (FDR = 4.58E-15), and other signal pathways (**Table 1**). The functional analysis showed the enrichment of emodin-regulated genes on the HCC pathway (FDR = 3.78E-03) (**Table 2**).

**Table 1:** The KEGG enriched pathways involved in FDX1, GPX4 and SLC7A11.

KEGG ID	Description	False discovery rate (FDR)
mmu00480	Glutathione metabolism	1.70E-43
mmu04216	Ferroptosis	1.26E-28
mmu01100	Metabolic pathways	3.44E-23
mmu00982	Drug metabolism - cytochrome P450	9.27E-18
mmu00980	Metabolism of xenobiotics by cytochrome P450	1.03E-17
mmu01524	Platinum drug resistance	2.56E-17
mmu00983	Drug metabolism - other enzymes	1.03E-16
mmu05204	Chemical carcinogenesis	2.80E-16
mmu05418	Fluid shear stress and atherosclerosis	4.87E-16
mmu05225	Hepatocellular carcinoma	4.58E-15
mmu05200	Pathways in cancer	8.14E-10
mmu00430	Taurine and hypotaurine metabolism	3.98E-06
mmu00590	Arachidonic acid metabolism	7.11E-06
mmu04978	Mineral absorption	1.35E-05
mmu00270	Cysteine and methionine metabolism	1.06E-02
mmu00450	Selenocompound metabolism	2.80E-02

**Table 2:** The KEGG enriched pathways involved in emodin.

KEGG ID	Description	False discovery rate (FDR)
mmu04110	Cell cycle	9.98E-06
mmu05161	Hepatitis B	9.98E-06
mmu05135	Yersinia infection	1.22E-05
mmu05223	Non-small cell lung cancer	1.26E-05
mmu05152	Tuberculosis	1.94E-04
mmu05212	Pancreatic cancer	2.83E-04
mmu04380	Osteoclast differentiation	2.83E-04
mmu04662	B cell receptor signaling pathway	3.70E-04
mmu04668	TNF signaling pathway	4.79E-04
mmu04115	p53 signaling pathway	5.12E-04
mmu05162	Measles	5.12E-04
mmu05133	Pertussis	6.94E-04
mmu05235	PD-L1 expression and PD-1 checkpoint pathway in cancer	8.23E-04
mmu05418	Fluid shear stress and atherosclerosis	8.23E-04
mmu05214	Glioma	9.36E-04
mmu04625	C-type lectin receptor signaling pathway	9.36E-04
mmu05222	Small cell lung cancer	9.36E-04
mmu04722	Neurotrophin signaling pathway	9.51E-04
mmu04620	Toll-like receptor signaling pathway	1.08E-03
mmu05168	Herpes simplex virus 1 infection	1.09E-03
mmu03420	Nucleotide excision repair	1.09E-03
mmu05164	Influenza A	1.12E-03
mmu05205	Proteoglycans in cancer	1.16E-03
mmu04926	Relaxin signaling pathway	1.16E-03
mmu04933	AGE-RAGE signaling pathway in diabetic complications	1.20E-03
mmu05163	Human cytomegalovirus infection	1.41E-03
mmu04922	Glucagon signaling pathway	1.41E-03
mmu01212	Fatty acid metabolism	1.41E-03
mmu04152	AMPK signaling pathway	1.41E-03
mmu00240	Pyrimidine metabolism	1.41E-03
mmu03013	RNA transport	1.41E-03
mmu00330	Arginine and proline metabolism	1.41E-03
mmu04919	Thyroid hormone signaling pathway	1.60E-03
mmu03030	DNA replication	1.90E-03
mmu04146	Peroxisome	1.90E-03
mmu00450	Selenocompound metabolism	1.91E-03
mmu05134	Legionellosis	1.92E-03
mmu00280	Valine, leucine and isoleucine degradation	1.96E-03
mmu04120	Ubiquitin mediated proteolysis	2.12E-03
mmu05220	Chronic myeloid leukemia	2.12E-03
mmu05132	Salmonella infection	2.37E-03
mmu00310	Lysine degradation	2.57E-03
mmu04070	Phosphatidylinositol signaling system	2.57E-03
mmu04010	MAPK signaling pathway	3.01E-03
mmu05167	Kaposi sarcoma-associated herpesvirus infection	3.05E-03
mmu00900	Terpenoid backbone biosynthesis	3.10E-03
mmu05215	Prostate cancer	3.10E-03
mmu05225	Hepatocellular carcinoma	3.78E-03
mmu04062	Chemokine signaling pathway	4.22E-03
mmu05140	Leishmaniasis	4.57E-03
mmu04935	Growth hormone synthesis, secretion and action	4.57E-03
mmu04218	Cellular senescence	5.22E-03
mmu04931	Insulin resistance	5.31E-03
mmu05169	Epstein-Barr virus infection	5.31E-03
mmu05210	Colorectal cancer	5.37E-03
mmu04150	mTOR signaling pathway	6.09E-03
mmu04659	Th17 cell differentiation	6.12E-03
mmu04630	JAK-STAT signaling pathway	6.12E-03
mmu01524	Platinum drug resistance	6.39E-03
mmu04657	IL-17 signaling pathway	6.39E-03
mmu01521	EGFR tyrosine kinase inhibitor resistance	9.25E-03
mmu04068	FoxO signaling pathway	1.01E-02
mmu04350	TGF-beta signaling pathway	1.05E-02
mmu00620	Pyruvate metabolism	1.06E-02
mmu00062	Fatty acid elongation	1.07E-02
mmu04666	Fc gamma R-mediated phagocytosis	1.23E-02
mmu04151	PI3K-Akt signaling pathway	1.34E-02
mmu04114	Oocyte meiosis	1.41E-02
mmu05145	Toxoplasmosis	1.43E-02
mmu04211	Longevity regulating pathway	1.49E-02
mmu04912	GnRH signaling pathway	1.49E-02
mmu04914	Progesterone-mediated oocyte maturation	1.49E-02
mmu00100	Steroid biosynthesis	1.49E-02
mmu03430	Mismatch repair	1.53E-02
mmu05211	Renal cell carcinoma	1.53E-02
mmu00920	Sulfur metabolism	1.53E-02
mmu00230	Purine metabolism	1.53E-02
mmu00640	Propanoate metabolism	1.53E-02
mmu00630	Glyoxylate and dicarboxylate metabolism	1.54E-02
mmu01523	Antifolate resistance	1.54E-02
mmu04540	Gap junction	1.54E-02
mmu00071	Fatty acid degradation	1.59E-02
mmu01522	Endocrine resistance	1.59E-02
mmu03018	RNA degradation	1.59E-02
mmu05166	Human T-cell leukemia virus 1 infection	1.60E-02
mmu04810	Regulation of actin cytoskeleton	1.63E-02
mmu04371	Apelin signaling pathway	1.69E-02
mmu04510	Focal adhesion	1.79E-02
mmu05202	Transcriptional misregulation in cancer	1.93E-02
mmu04015	Rap1 signaling pathway	2.10E-02
mmu05221	Acute myeloid leukemia	2.39E-02
mmu04910	Insulin signaling pathway	2.43E-02
mmu04623	Cytosolic DNA-sensing pathway	2.66E-02
mmu04664	Fc epsilon RI signaling pathway	2.66E-02
mmu00270	Cysteine and methionine metabolism	2.87E-02
mmu04920	Adipocytokine signaling pathway	3.04E-02
mmu04611	Platelet activation	3.08E-02
mmu04210	Apoptosis	3.21E-02
mmu04370	VEGF signaling pathway	3.34E-02
mmu04710	Circadian rhythm	3.44E-02
mmu05203	Viral carcinogenesis	3.46E-02
mmu00790	Folate biosynthesis	3.62E-02
mmu00562	Inositol phosphate metabolism	3.70E-02
mmu05218	Melanoma	3.70E-02
mmu04360	Axon guidance	3.70E-02
mmu04650	Natural killer cell mediated cytotoxicity	3.99E-02
mmu04066	HIF-1 signaling pathway	3.99E-02
mmu00510	N-Glycan biosynthesis	4.04E-02
mmu03320	PPAR signaling pathway	4.50E-02
mmu04660	T cell receptor signaling pathway	4.50E-02
mmu05017	Spinocerebellar ataxia	4.65E-02
mmu04658	Th1 and Th2 cell differentiation	5.92E-02
mmu00670	One carbon pool by folate	6.08E-02
mmu01230	Biosynthesis of amino acids	6.22E-02
mmu04530	Tight junction	6.43E-02
mmu01040	Biosynthesis of unsaturated fatty acids	6.43E-02
mmu04622	RIG-I-like receptor signaling pathway	6.43E-02
mmu05160	Hepatitis C	6.51E-02
mmu04064	NF-kappa B signaling pathway	6.61E-02
mmu05170	Human immunodeficiency virus 1 infection	6.69E-02
mmu04640	Hematopoietic cell lineage	6.74E-02
mmu00520	Amino sugar and nucleotide sugar metabolism	7.13E-02
mmu05034	Alcoholism	7.26E-02
mmu04670	Leukocyte transendothelial migration	7.44E-02
mmu04728	Dopaminergic synapse	7.65E-02
mmu04217	Necroptosis	7.88E-02
mmu00561	Glycerolipid metabolism	7.95E-02
mmu05321	Inflammatory bowel disease	7.95E-02
mmu04140	Autophagy - animal	8.16E-02
mmu01210	2-Oxocarboxylic acid metabolism	8.74E-02
mmu03460	Fanconi anemia pathway	8.79E-02
mmu05016	Huntington disease	9.06E-02
mmu00410	beta-Alanine metabolism	9.21E-02
mmu03020	RNA polymerase	9.21E-02
mmu05142	Chagas disease	9.21E-02
mmu04012	ErbB signaling pathway	9.21E-02
mmu00533	Glycosaminoglycan biosynthesis - keratan sulfate	9.21E-02
mmu04216	Ferroptosis	9.21E-02
mmu04928	Parathyroid hormone synthesis, secretion and action	9.39E-02
mmu04330	Notch signaling pathway	9.89E-02
mmu04621	NOD-like receptor signaling pathway	1.00E-01
mmu04141	Protein processing in endoplasmic reticulum	1.04E-01
mmu05010	Alzheimer disease	1.04E-01

### Emodin promotes cuproptosis in HCCLM3 cells

**Figure 3:**
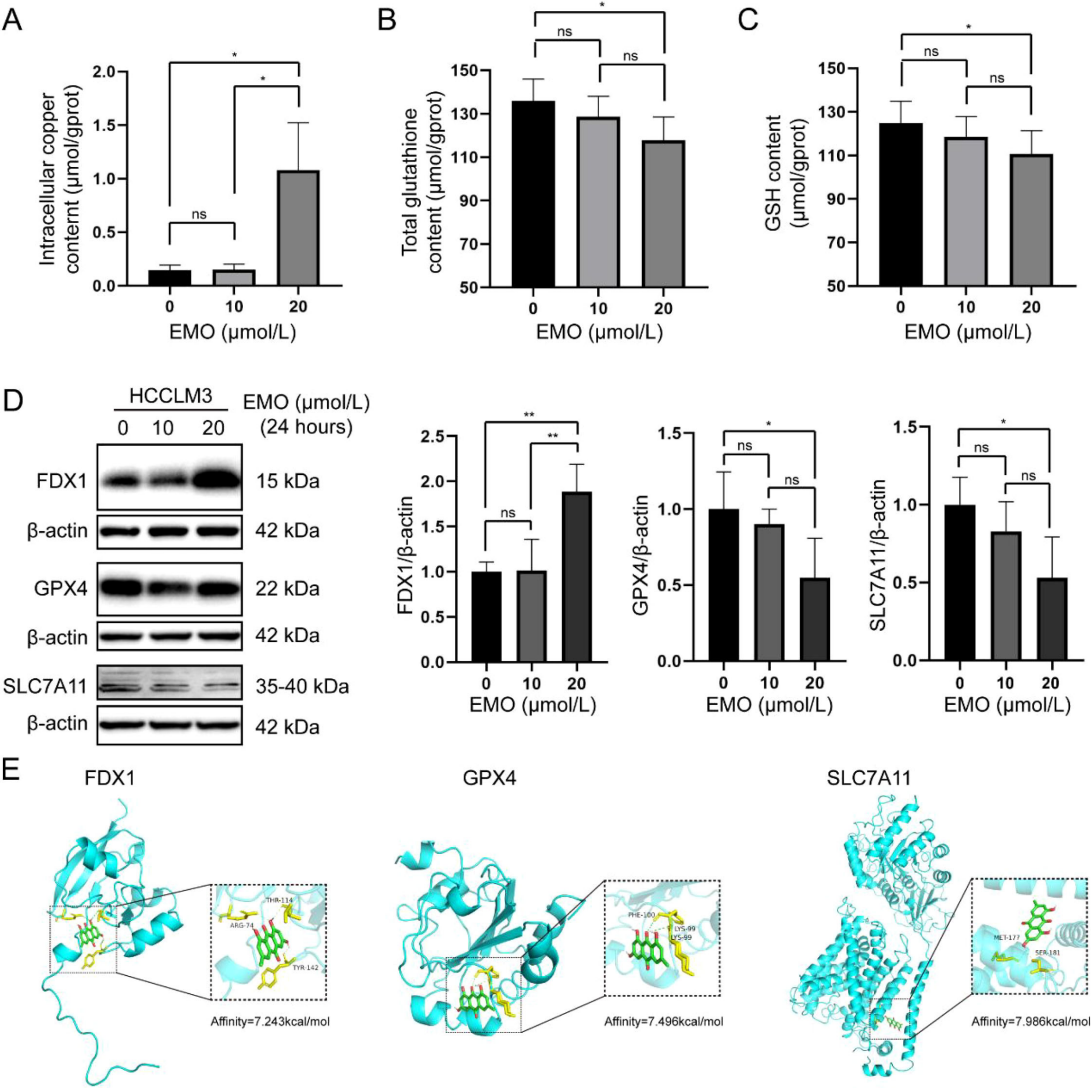
Emodin induced HCCLM3 cells cuproptosis. Intracellular copper content (n=3) **(A)**, total glutathione (n=5) **(B)** and GSH (n=5) **(C)** content in HCCLM3 cells after intervention with different concentrations of emodin for 24 (h) (n=3) **(D)** Representative image of western blot results of HCCLM3 cells (left) and the charts of semi-quantitative statistical results. ns, *P* > 0.05, *P < 0.05, **P < 0.01. **(E)** Molecular docking diagram of FDX1 (Affinity=7.243kcal/mol), GPX4 (Affinity=7.496kcal/mol) and SLC7A11 (Affinity=7.986kcal/mol) proteins and emodin. The green molecule is emodin.

Building on database analyses, validation in HCCLM3 cells revealed that emodin treatment significantly increased intracellular copper ion levels at 20 μmol/L (**Figure 3A**), suggesting cuproptosis induction, while dose-dependently upregulating FDX1 expression and downregulating GPX4 and SLC7A11 (**Figure 3D**), accompanied by reduced total glutathione and GSH levels (**Figures 3B, C**). Molecular docking demonstrated emodin’s binding to SLC7A11, GPX4, and FDX1 (**Figure 3E**), indicating multi-target effects that disrupt copper homeostasis by decreasing GSH synthesis, elevating free Cu^2+^, suppressing GPX4, and enhancing FDX1-mediated copper toxicity, collectively promoting cuproptosis via the SLC7A11/FDX1 axis.

### Emodin suppresses subcutaneous HCC tumor growth in nude mice

**Figure 4:**
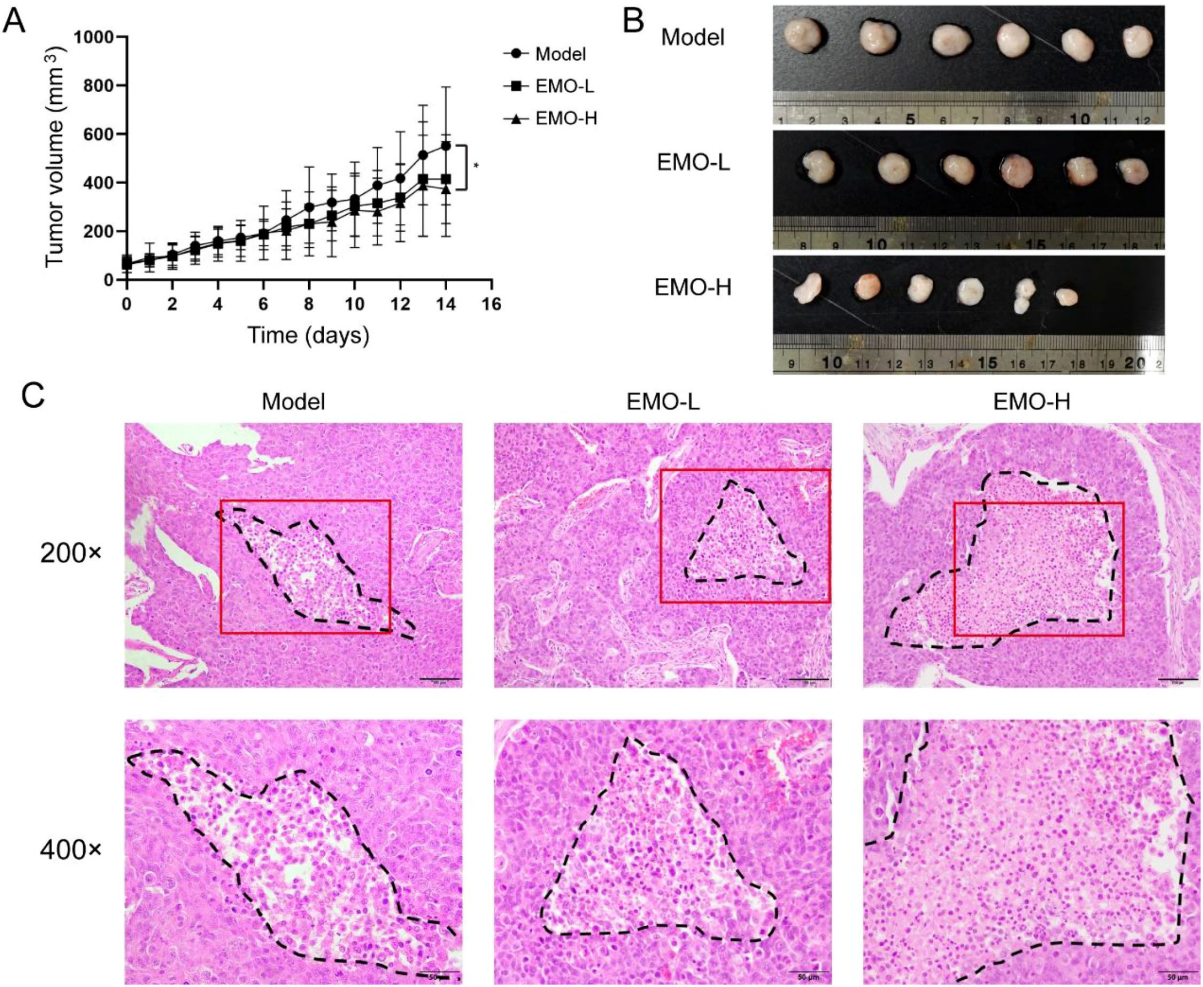
Emodin inhibited HCC subcutaneous transplantation tumor growth in nude mice. **(A)** Curve of tumor volume change of nude mice in model (n=10), emodin low-dose (EMO-L, n=10) group and emodin high-dose (EMO-H, n=10) group. **(B)** Images of tumor in different groups. **(C)** Representative images of tumor tissues H&E staining for different groups, dotted line showed the necrotic area. *P < 0.05.

To further confirm whether emodin exerts its anti-tumor effect via induction of cuproptosis, we established a subcutaneous xenograft model using HCCLM3 cells in nude mice. Compared with the model group, mice treated with emodin showed significantly smaller tumor volumes, with the high-dose group exhibiting greater tumor growth inhibition (**Figures 4A, B**). We have statistically analyzed the number of positive cells in four 400× random fields based on IHC staining results (**Supplementary Table 3**). Histological examination by H&E staining revealed expanded areas of coagulative necrosis (characterized by eosinophilic cytoplasm and nuclear loss) in the emodin-treated groups, which suggests that the cell death is associated with cuproptosis. In contrast, non-necrotic tumour areas maintained intact nuclear membranes (**Figure 4C**).

### Emodin enhances cuproptosis levels in tumor tissues

**Figure 5:**
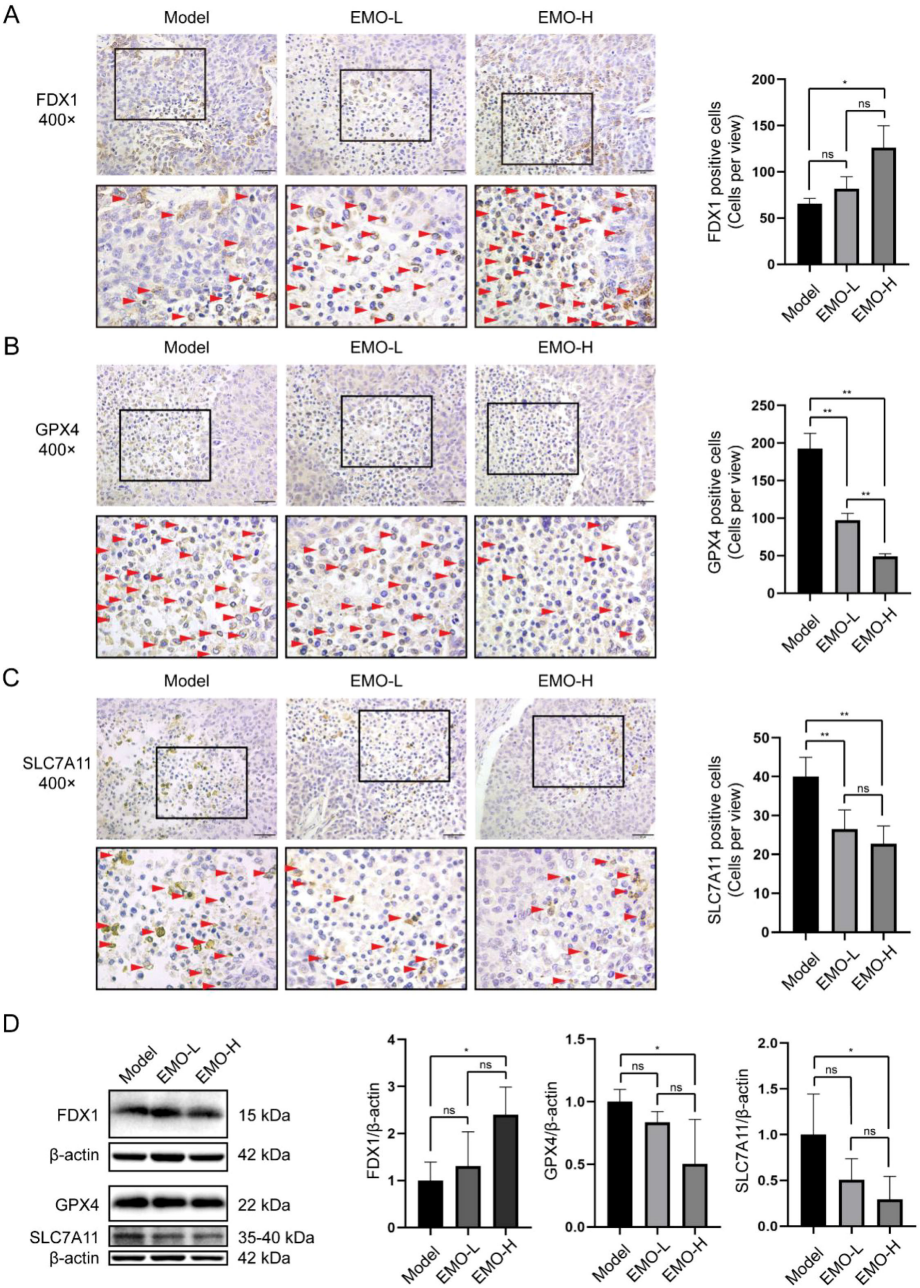
Emodin elevated the level of cuproptosis in HCC tumor tissues. Representative images of immunohistochemistry staining from model, emodin low-dose (EMO-L), emodin high-dose (EMO-H) group and statistics charts for count of positive cells for FDX1 **(A)**, GPX4 **(B)** and SLC7A11 **(C)**, the number of positive cells were counted and statistics at least 4 views of 400x random field. Red arrows showed the positive cells (n=4). **(D)** Representative image of western blot results of tumor tissues from different groups (left), and the charts of semi-quantitative statistical results (n=6). ns, *P* > 0.05, ^*^P < 0.05, ^**^P < 0.01.

To verify whether emodin induces cuproptosis *in vivo*, immunohistochemical staining for FDX1, GPX4, and SLC7A11 was performed on tumor tissue sections. FDX1-positive staining was primarily localized to necrotic areas and increased with higher emodin doses (**Figure 5A**). Conversely, GPX4- and SLC7A11-positive cells were reduced in the high-dose group compared to the model group (**Figures 5B, C**). Western blot analysis further confirmed these findings. FDX1 expression increased in a dose-dependent manner, while GPX4 and SLC7A11 expression decreased accordingly (**Figure 5D**). These data indicate that emodin treatment enhances cuproptosis levels in HCC tumor tissues *in vivo*.

### FDX1 knockdown rescues emodin-induced cuproptosis phenotypes

**Figure 6:**
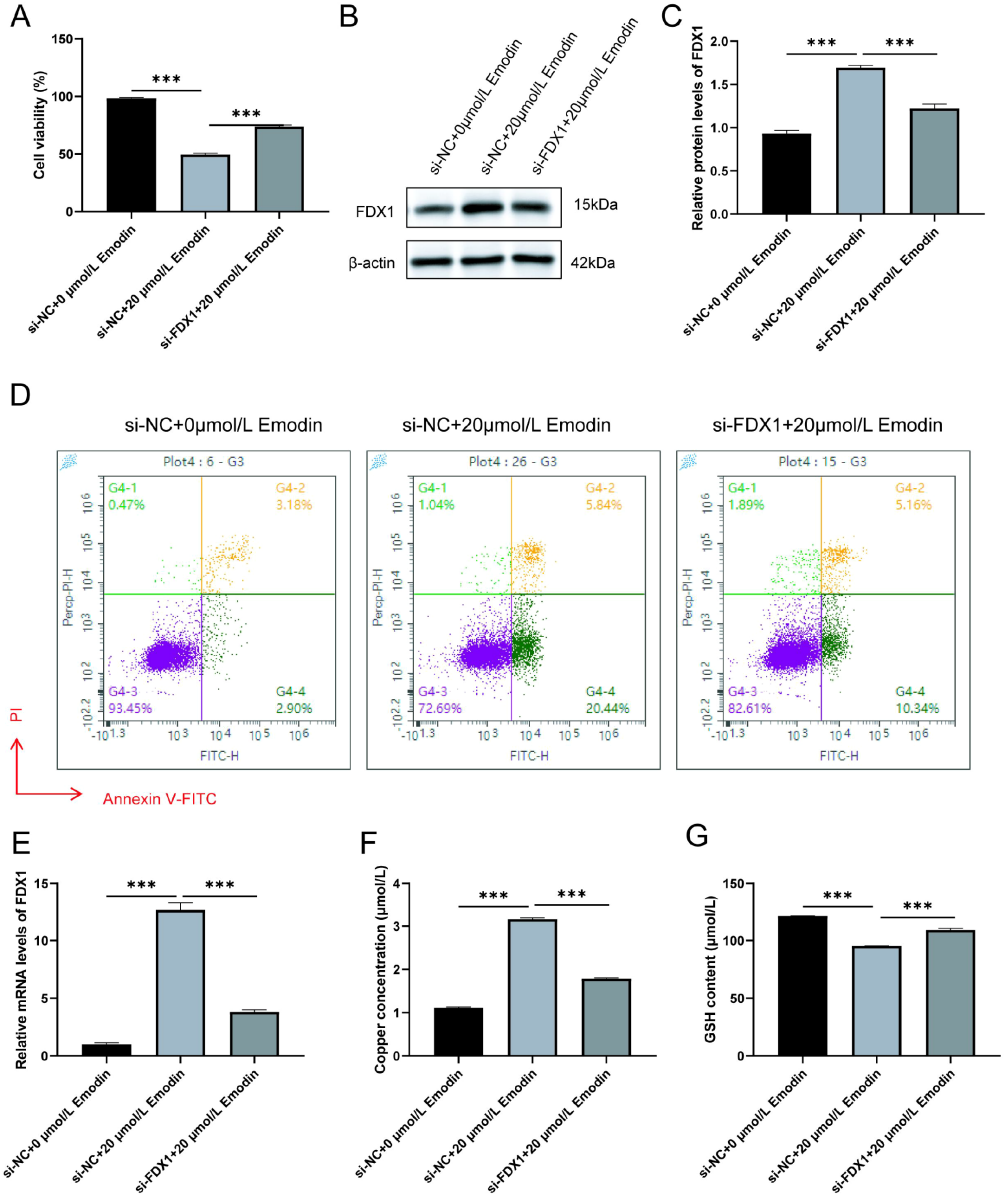
FDX1 knockdown rescues emodin-induced cuproptosis phenotypes in HCCLM3 cells. **(A)** RT-qPCR analysis of FDX1 mRNA expression in si-NC and si-FDX1 transfected cells. **(B)** Representative Western blot image of FDX1 protein expression.**(C)** Quantification of FDX1 protein levels from **(B)**. **(D)** Cell viability assessed by CCK-8 assay after treatment with or without emodin (20 μM) for 24 h. **(E)** Representative flow cytometry plots of apoptosis (Annexin V/PI staining). **(F)** Quantification of total apoptosis rate from **(E)**. **(G)** Intracellular copper ion concentration. **(H)** Intracellular GSH content. All data are presented as mean ± SD (n = 3). *P < 0.05, **P < 0.01, ***P < 0.001; ns, not significant (one-way ANOVA with Tukey’s *post hoc* test).

To establish a direct causal role for FDX1 in emodin’s mechanism, we performed FDX1 knockdown in HCCLM3 cells. Transfection with si-FDX1 successfully reduced both FDX1 mRNA and protein expression compared to the si-NC control (**Figures 6A–C**). In these FDX1-deficient cells, the cytotoxic effect of emodin (20 μM) was significantly attenuated, with cell viability markedly restored (**Figure 6D**). Accordingly, the emodin-induced increase in apoptosis observed in control cells was also blunted in FDX1-KD cells (**Figures 6E, F**). Crucially, the hallmark biochemical events of cuproptosis were reversed: the emodin-triggered accumulation of intracellular copper ions was completely abrogated (**Figure 6G**), and the depletion of glutathione (GSH) was significantly rescued (**Figure 6H**). These data demonstrate that FDX1 is essential for emodin to induce copper overload, antioxidant collapse, and subsequent cell death, providing direct genetic evidence that emodin triggers cuproptosis specifically via an FDX1-dependent pathway.

### Pharmacological inhibition confirms cuproptosis as the specific cell death pathway

To definitively establish that emodin-induced cytotoxicity is specifically due to cuproptosis and not other common regulated cell death pathways, we performed rescue experiments using specific pharmacological inhibitors. Co-treatment with the copper chelator tetrathiomolybdate (TTM), a known inhibitor of cuproptosis, significantly attenuated the reduction in cell viability caused by emodin (20 μM) (**Supplementary Figure 2A**). In contrast, co-treatment with the potent ferroptosis inhibitor ferrostatin-1 (Fer-1) or the pan-caspase apoptosis inhibitor Z-VAD-FMK did not rescue cell viability (**Supplementary Figure 2A**). Concordantly, flow cytometric analysis of apoptosis revealed that the increase in apoptotic cells induced by emodin was specifically reversed by TTM but not by Fer-1 or Z-VAD-FMK (**Supplementary Figures 2B, C**). These data demonstrate that emodin-induced cell death is specifically dependent on copper ion availability and is distinct from ferroptosis and apoptosis under these experimental conditions, providing strong pharmacological evidence that emodin triggers cuproptosis in HCCLM3 cells.

## Discussion

Our findings demonstrate that emodin can induce cuproptosis in HCC cells by modulating the SLC7A11/FDX1 axis, offering new insights into its anti-tumor mechanism. As a newly characterized form of programmed cell death, cuproptosis is primarily driven by the accumulation of intracellular copper ions, leading to lipoylated protein aggregation and proteotoxic stress (5). We selected the HCCLM3 cell line for *in vitro* and *in vivo* validation due to its highly aggressive and metastatic characteristics, which represent a challenging subtype of HCC with limited treatment options (12). By demonstrating the efficacy of emodin in inhibiting such an aggressive model, our findings highlight its potential therapeutic value for advanced HCC. In this study, emodin treatment downregulated SLC7A11 expression, impaired glutathione (GSH) synthesis, reduced GPX4 activity, and weakened copper chelation capacity. Simultaneously, FDX1 expression was upregulated, suggesting a dual mechanism through which emodin promotes cuproptosis-namely, inhibition of the GSH-GPX4 antioxidant system and activation of FDX1-mediated copper toxicity.

SLC7A11, a core component of the cystine/glutamate antiporter system Xc⁻, plays a critical role in cystine uptake and GSH biosynthesis (12). Our data showed that emodin reduced SLC7A11 expression in a dose-dependent manner, consistent with previous findings on SLC7A11 inhibitors such as erastin, which also trigger cuproptosis (13). Recent advances in cuproptosis research across various cancer types further support our findings. Li et al. demonstrated that a copper-based silk fibroin nanoplatform could induce cuproptosis in metastatic breast cancer by promoting DLAT oligomerization, achieving synergistic effects through photothermal and chemodynamic therapy combined with immune activation (14). Similarly, Sun et al. developed GSH/pH-responsive copper-based nanocomplexes that triggered cuproptosis alongside ferroptosis and necroptosis in oral squamous cell carcinoma, depleting glutathione and disrupting mitochondrial function to achieve 92.3% tumor inhibition (15). These studies, together with other emerging evidence (3, 16), underscore the growing recognition of cuproptosis as a viable therapeutic target across multiple malignancies, reinforcing the translational potential of emodin-induced cuproptosis in HCC. Notably, inhibition of SLC7A11 not only limits cystine availability precursor for GSH synthesis-but also disrupts cellular redox homeostasis by reprogramming GSH metabolism (17, 18). Furthermore, emodin-mediated downregulation of GPX4 implies that disruption of the GSH-GPX4 axis is a pivotal event in the induction of cuproptosis. FDX1, a mitochondrial reductase involved in copper metabolism, may act as a central regulator of cuproptosis through two mechanisms: by reducing Cu²^+^ to the more toxic Cu^+^, thereby exacerbating lipoylated protein damage (19); and by participating in mitochondrial respiration through regulation of Fe-S cluster assembly (20). We observed that emodin treatment significantly increased FDX1 expression in a dose-dependent manner, supporting previous studies showing that FDX1 knockdown impairs cuproptosis (21). This indicates that FDX1 may serve as a key amplifier in the cuproptosis pathway.

Importantly, our results also suggest that emodin may reshape the copper metabolic network in HCC cells by simultaneously downregulating GSH and upregulating FDX1, forming a vicious cycle of “enhanced oxidative stress and weakened antioxidant defense” (22–27). The dual effects of emodin may converge on the Keap1-Nrf2 pathway, which inhibits Nrf2 nuclear translocation. Depletion of glutathione blocks Keap1 sulfhydryl modification, resulting in Nrf2 remaining in an inhibited state and establishing a self-amplifying cycle of copper toxicity (28). These mechanisms merit further investigation. Furthermore, the frequent amplification of the SLC7A11 gene in hepatocellular carcinoma tissues indicates that emodin may have significant therapeutic potential for subtypes of HCC that exhibit high SLC7A11 expression (29, 30). The main advantages of emodin are its ability to guide therapy using biomarkers, reverse immunosuppression by modulating chemokines, enhance antitumor efficacy in combination with other therapeutic agents, and overcome resistance associated with single-target interventions.

However, this study has several limitations. Firstly, the *in vitro* experiments used only one hepatocellular carcinoma cell line and therefore require validation in other liver cancer cell models. Secondly, validation of the FDX1 gene knockout remains insufficient and further genetic manipulation is required to confirm causality. Thirdly, the study did not explore the mechanistic role of emodin metabolites. When translating dosages from the current animal study to clinical settings, the challenge of excessive dosing may arise due to insufficient tumour targeting. Future work should therefore focus on optimizing delivery methods to enhance tumour specificity. Additionally, while no emodin-related weight loss was observed in this study, the long-term systemic effects remain to be investigated. Future studies employing tumour organoid models, single-cell RNA sequencing and other advanced technologies may elucidate the multidimensional regulatory network of emodin within the HCC microenvironment (31, 32).

In conclusion, emodin appears to induce cuproptosis in HCC cells by downregulating SLC7A11 expression, reducing intracellular GSH levels, impairing copper ion chelation, increasing intracellular copper accumulation, and upregulating FDX1 expression. These findings provide new mechanistic insights into the anti-cancer activity of emodin and may support its further development as a therapeutic agent for HCC.

## Data Availability

The original contributions presented in the study are included in the article/**Supplementary Material**. Further inquiries can be directed to the corresponding author.
