# Supplementary figures and images for: Emodin triggers cuproptosis to suppress hepatocellular carcinoma via SLC7A11/FDX1 axis
Source: Front Oncol. 2026 Mar 25;16:1756712. doi: 10.3389/fonc.2026.1756712 (PMC13056838; doi:10.3389/fonc.2026.1756712)

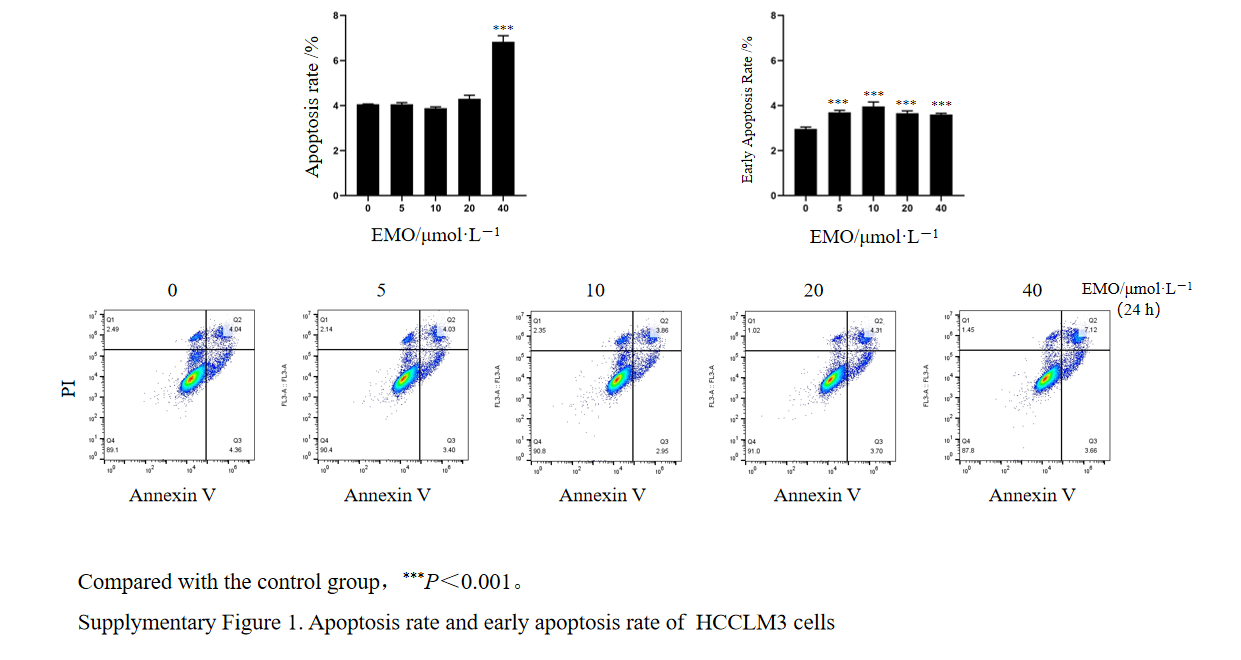

Supplement: Supplementary file 1 [file Image1.png]

A

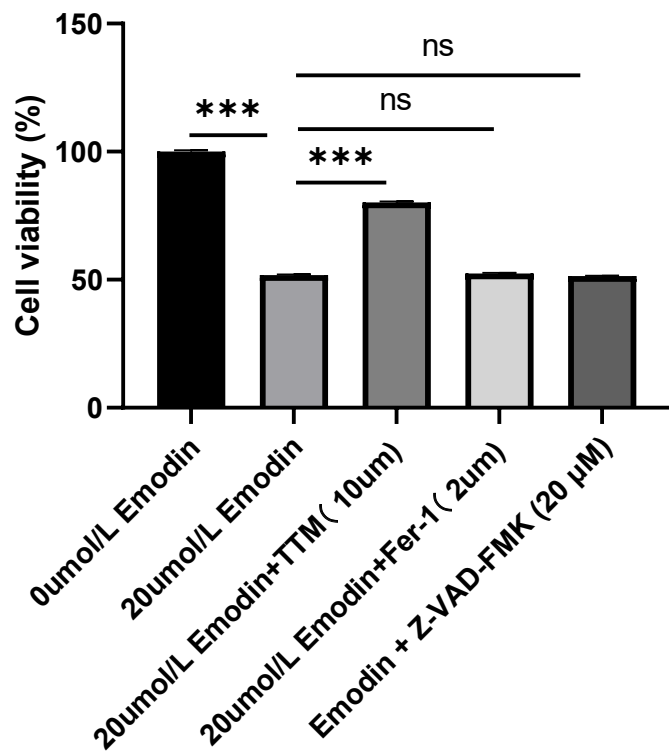

B

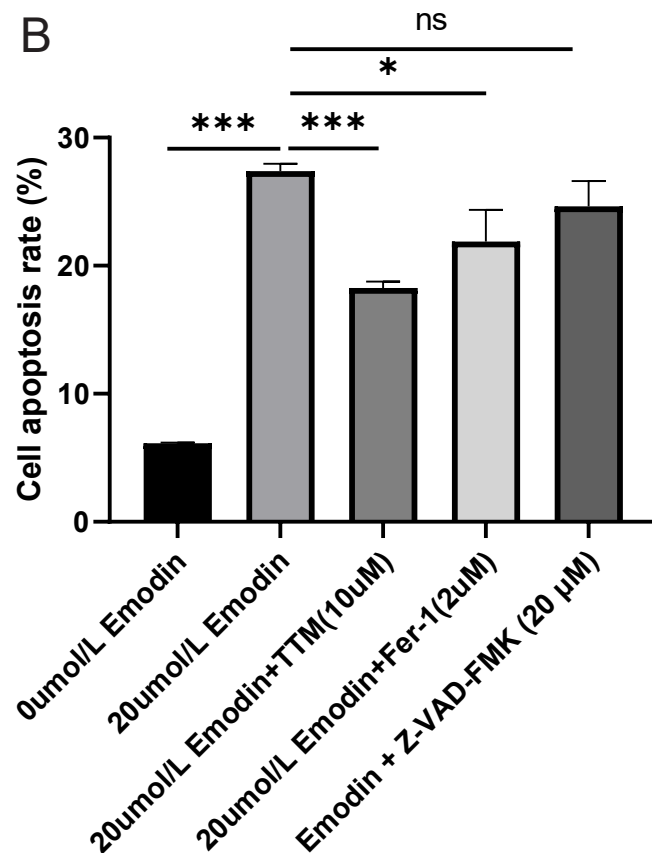

C

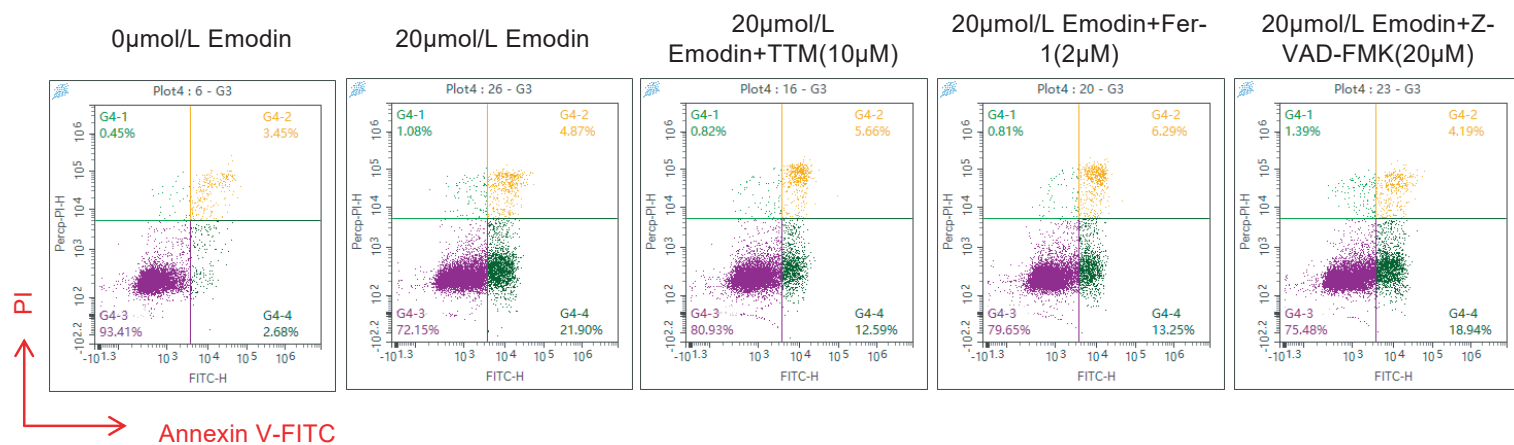

Supplement: Supplementary file 2 [file Image2.pdf]
